# Synergistic Triplet Exciton Management and Interface Engineering for High-Brightness Sky-Blue Multi-Cation Perovskite Light-Emitting Diodes

**DOI:** 10.3390/nano16010004

**Published:** 2025-12-19

**Authors:** Fawad Ali, Fang Yuan, Shuaiqi He, Peichao Zhu, Nabeel Israr, Songting Zhang, Puyang Wu, Jiaxin Liang, Wen Deng, Zhaoxin Wu

**Affiliations:** 1 Key Laboratory for Physical Electronics and Devices of the Ministry of Education & Shaanxi Key Lab of Information Photonic Technique, School of Electronic Science and Engineering, Xi’an Jiaotong University, Xi’an 710049, China; fawad_ali@stu.xjtu.edu.cn (F.A.);; 2 Journal Editorial Department, Xi’an Jiaotong University, Xi’an 710065, China; 3 Collaborative Innovation Center of Extreme Optics, Shanxi University, Taiyuan 030006, China

**Keywords:** blue perovskite light-emitting diodes, multi-cation perovskite films, triplet exciton management, interface engineering

## Abstract

Perovskite light-emitting diodes (PeLEDs) have garnered significant interest owing to their exceptional color purity, broadly tunable emission spectra, and cost-effective solution processability. However, blue PeLEDs continue to underperform in efficiency and operational stability compared to their red and green counterparts, primarily due to defect-induced non-radiative recombination losses and inefficient exciton management. Herein, we demonstrate a synergistic approach that integrates multi-cation compositional engineering with triplet exciton management by incorporating a high-triplet-energy material, mCBP (3,3-Di(9H-carbazol-9-yl)biphenyl), during film fabrication. Temperature-dependent photoluminescence reveals that mCBP incorporation significantly enhances the exciton binding energy from 49.36 meV to 68.84 meV and reduces phonon coupling strength, indicating improved exciton stability and suppressed non-radiative channels. The corresponding PeLEDs achieve a peak external quantum efficiency of 10.2% and a maximum luminance exceeding 12,000 cd/m^2^, demonstrating the effectiveness of this solution-based triplet management strategy. This work highlights the critical role of scalable, solution-processed triplet exciton management strategies in advancing blue PeLED performance, offering a practical pathway toward high-performance perovskite-based display and lighting technologies.

## 1. Introduction

Metal halide perovskite light-emitting diodes (PeLEDs) have emerged as a disruptive technology in the field of optoelectronics, capturing significant interest owing to their exceptional color purity with narrow emission linewidths, broadly tunable emission spectra spanning from the deep-blue to the near-infrared region, and excellent solution processability that enables low-cost, scalable fabrication techniques such as spin-coating and inkjet printing [1,2,3,4,5,6,7,8]. These compelling characteristics position PeLEDs as promising candidates for next-generation applications in high-resolution, wide-color-gamut displays and energy-efficient solid-state lighting technology, where achieving a balance between high performance, stability, and manufacturing scalability is crucial [9,10,11]. Over the past decade, remarkable progress has been achieved in red and green PeLEDs, with external quantum efficiencies (EQEs) exceeding 30%, values that are approaching the theoretical limits imposed by light out-coupling efficiency [7,11,12,13,14]. Beyond light-emitting applications, metal halide perovskites have demonstrated remarkable versatility across diverse optoelectronic domains, including high-efficiency photovoltaics, sensitive photodetectors, low-threshold lasers, and emerging memory devices [15,16]. This broad applicability stems from the unique combination of defect-tolerant electronic structure, tunable bandgaps via compositional engineering, and solution processability at low temperatures.

In stark contrast, the development of efficient and stable blue PeLEDs has proven substantially more challenging, resulting in a conspicuous ‘blue gap’ in the perovskite optoelectronics landscape. Blue emission is indispensable for realizing full-color displays, where it serves as one of the three primary colors (red, green, and blue, or RGB), and for generating high-quality white light in lighting technologies, typically through color mixing with yellow phosphors or other emissive materials. Despite their critical importance, blue PeLEDs have made significant progress in recent years, with reported EQEs now exceeding 20% in state-of-the-art devices [17,18]. However, challenges remain in achieving a combination of high efficiency, excellent color purity, and robust operational stability simultaneously. This persistent performance gap not only hinders the realization of all-perovskite white LEDs and displays but also poses a major obstacle to the commercialization and market adoption of full-color perovskite-based display technologies [7,14,18,19,20].

The inferior performance of blue PeLEDs stems from multifaceted challenges, including intrinsic material properties and interfacial issues. To achieve the wider bandgaps necessary for blue emission, compositional engineering often involves increasing the chlorine-to-bromide ratio or reducing dimensionality, which tends to accelerate crystallization kinetics during film formation. This rapid crystallization frequently results in poor surface morphology, numerous grain boundaries, and the formation of halide vacancies and lead-related deep-level defects (e.g., Pb^0^, Pb clusters) [21,22,23,24]. These defects act as strong non-radiative recombination centers, significantly reducing the photoluminescence quantum yield (PLQY) of the emissive layer. Furthermore, the wider bandgap of blue-emitting perovskites often leads to significant energy-level misalignment with the highest occupied molecular orbital (HOMO) and lowest unoccupied molecular orbital (LUMO) levels of adjacent charge transport layers (CTLs), causing inefficient charge injection, unbalanced carrier transport, and accumulation of charges at the interfaces [11,25,26,27,28]. Such issues collectively contribute to high leakage currents, low radiative recombination rates, efficiency roll-off at practical operating currents, and limited operational stability, often manifesting as rapid degradation under electrical bias for blue PeLED devices [29,30,31,32,33,34,35,36,37].

To address these challenges, a variety of innovative strategies have been explored to enhance the performance of blue PeLEDs. These include multi-cation compositional engineering (e.g., incorporating Cs^+^, FA^+^, MA^+^, Rb^+^, and K^+^) to improve crystallinity and phase stability; quasi-2D/3D heterostructure engineering to achieve efficient energy funneling and confinement; advanced defect passivation techniques using organic or inorganic ligands (e.g., phenethylammonium bromide and potassium halides); and interfacial modification with suitable interlayers to improve charge injection balance [5,28,30,38,39,40,41,42]. For example, Yuan et al. have demonstrated that multi-cation perovskite compositions, specifically incorporating FA^+^, Rb^+^, and K^+^, can significantly enhance film uniformity, suppress ion migration, and improve blue emission characteristics [12,43]. While these approaches have led to incremental improvements in film quality and device metrics, the overall performance of blue PeLEDs remains unsatisfactory. This suggests that additional, more nuanced loss mechanisms, particularly those related to exciton dynamics and quenching at the critical interfaces between the perovskite and CTLs, require further investigation and mitigation.

One critical yet often overlooked loss pathway is the presence of long-lived triplet excitons, which can undergo non-radiative recombination at the perovskite/CTLs interfaces [44]. In perovskite semiconductors, spin–orbit coupling can facilitate intersystem crossing, populating triplet exciton states. These triplet excitons, characterized by their relatively long diffusion lengths and lifetimes, can migrate to interfaces where they undergo non-radiative energy transfer to adjacent CTLs if their triplet energy is lower than that of the perovskite emitter. This process serves as a significant non-radiative recombination channel, depleting the exciton population available for radiative recombination. Recent studies have begun to address this issue by incorporating high-triplet-energy organic interlayers or additives, such as specific carbazole derivatives, which act as exciton-blocking layers. These layers help to confine triplet excitons within the emissive perovskite layer and block detrimental energy transfer to adjacent layers, thereby preserving the exciton population and enhancing device efficiency [45,46]. However, most previous efforts have focused either on bulk defect passivation or interfacial exciton management in isolation. Meanwhile, multi-cation engineering, involving the incorporation of a cocktail of A-site cations such as Cs^+^, FA^+^, and Rb^+^, has demonstrated considerable effectiveness in improving film quality, reducing bulk defects, and enhancing emission stability through strain relaxation and suppression of halide vacancy formation [47,48,49,50]. Despite these individual advances, the synergistic integration of multi-cation composition control aimed at mitigating bulk losses with deliberate triplet exciton management at the interfaces aimed at mitigating interfacial losses, has not been systematically explored in a unified strategy for high-performance blue PeLEDs. A holistic approach that concurrently addresses both bulk and interfacial loss mechanisms could potentially unlock new frontiers in blue PeLED performance.

In this work, we propose a synergistic strategy that seamlessly combines multi-cation perovskite composition engineering with targeted triplet exciton management at the critical perovskite/CTL interface. By introducing a high-triplet-energy organic small molecule, mCBP (3,3-Di(9H-carbazol-9-yl) biphenyl), into the anti-solvent during the film fabrication process, we simultaneously tackle multiple challenges: reducing lead-related vacancies and halide defects in the bulk, decreasing phonon scattering, improving interfacial energy level alignment, and, most importantly, suppressing triplet exciton quenching at the perovskite/CTL interface. X-ray photoelectron spectroscopy (XPS) confirms a significant reduction in lead-defect states, while time-resolved photoluminescence spectroscopy reveals markedly improved exciton dynamics and prolonged carrier lifetimes. As a result, the optimized sky-blue multi-cation perovskite films achieve a PLQY of 52%, representing a 37% improvement compared to untreated control films (38%). The corresponding PeLEDs based on mCBP-treated perovskite film exhibit a peak EQE of 10.2%, and a maximum luminance exceeding 12,000 cd/m^2^ at 486 nm. These results not only mark a meaningful advance toward efficient and stable blue PeLEDs but also offer valuable mechanistic insights into the critical role of integrated interfacial exciton management and bulk defect passivation in pushing the performance boundaries of perovskite-based optoelectronic devices.

## 2. Results and Discussion

**Figure 1:**
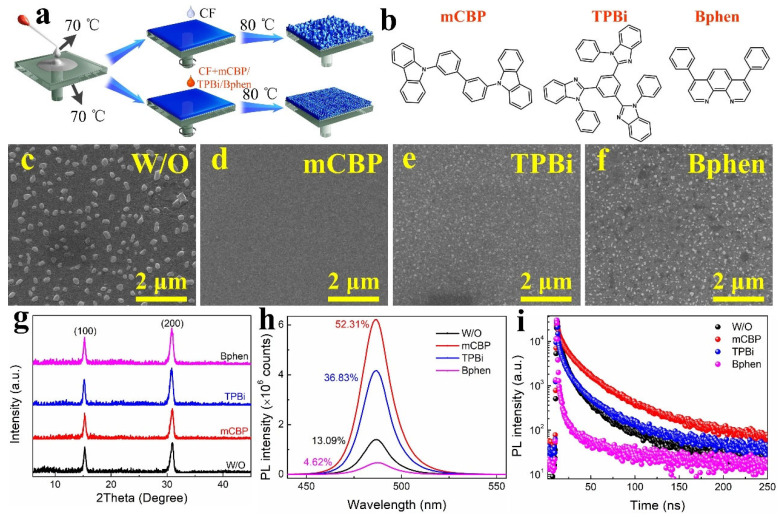
(**a**) Schematic illustration of the multi-cation perovskite film fabrication process using chloroform (CF) antisolvent with various small organic molecules (mCBP, TPBi, Bphen). (**b**) Molecular structures of mCBP, TPBi, and Bphen. (**c**–**f**) Top-view SEM images of perovskite films: (**c**) untreated (W/O), and treated with (**d**) mCBP, (**e**) TPBi, and (**f**) Bphen. (**g**) XRD patterns of the corresponding perovskite films, showing the (100) and (200) diffraction peaks of the cubic perovskite phase. (**h**) Steady-state photoluminescence (PL) spectra and corresponding photoluminescence quantum yields (PLQYs). (**i**) Time-resolved photoluminescence (TRPL) decay curves fitted with a bi-exponential function of different perovskite films.

We carefully engineered the composition of an all-inorganic CsPbBr_3−x_Cl_x_ perovskite film and introduced rubidium bromide (RbBr), potassium bromide (KBr), and phenethylamine bromide (PEABr) to effectively reduce grain size and passivate defects, building upon our previous work in multi-cation perovskite systems [4,12]. The composition of the luminescent layer (0.05 M Pb^2+^ in DMSO solvent) with PbBr_2_:CsCl:RbBr:PEABr:KBr in a molar ratio of 1:0.9:0.5:0.3:0.1, plays a crucial role in determining the optical properties of the films. The 3D multi-cation sky-blue (Cs/Rb/K/PEA)Pb(Br/Cl)_3_ perovskite films were fabricated via a one-step spin-coating process using chloroform (CHCl_3_, abbreviated as CF) as an anti-solvent, as schematically illustrated in Figure 1a. First, a perovskite precursor solution was prepared and deposited onto a substrate preheated to 70 °C to ensure rapid crystallization. Subsequently, the small organic molecules such as mCBP, TPBi, and Bphen were introduced via the antisolvent treatment. Finally, the films were annealed at 80 °C to crystallize the perovskite layer, a critical step for achieving the desired morphology and optoelectronic properties. The spin-coating process, chloroform antisolvent treatment, and thermal annealing at 80 °C for 20 min promote controlled crystallization, thereby enhancing film quality and reducing defects. Figure 1b displays the molecular structures of mCBP, TPBi, and Bphen, respectively. The PL images of perovskite films with various small organic molecule (mCBP, TPBi, Bphen) treatments under 365 nm UV light irradiation are shown in Figure S1, which shows that all the perovskite films emit uniformly bright sky-blue light.

The morphological evolution of the perovskite films was systematically investigated using top-view scanning electron microscopy (SEM). As shown in Figure 1c–f, the perovskite film fabricated without any small organic molecule treatment (W/O film) results in large, irregular grains with poor uniformity (Figure 1c). In stark contrast, the perovskite film treated with mCBP (Figure 1d) yields a densely packed film composed of smaller, uniformly distributed grains, indicating enhanced crystallinity and denser morphology. Similarly, the TPBi-treated perovskite film (Figure 1e) moderately refines grain alignment, likely due to its role in templating perovskite growth during deposition. However, the Bphen-treated perovskite film (Figure 1f) results in a less homogeneous morphology, suggesting a trade-off between charge transport optimization and structural uniformity. Furthermore, Figure S2 shows the mCBP-treated perovskite film’s elemental distribution, composition, and uniformity, confirming even incorporation of key components for high-quality films. These findings highlight the significant impact of small organic molecule treatment on the quality of perovskite film, which in turn correlates with key device performance metrics such as carrier mobility and radiative recombination efficiency. The visual appearance of the perovskite films under different lighting conditions further corroborates these morphological observations. As shown in Figure S3, under both incandescent and 365 nm UV illumination, the mCBP-treated films exhibit a more uniform and brighter sky-blue emission compared to the untreated film, indicating improved film quality and enhanced luminescent properties. To further understand the influence of these organic additives on the crystallization behavior of multi-cation perovskite films, X-ray diffraction (XRD) measurements were performed. As depicted in Figure 1g, there are characteristic diffraction peaks at 15.5° and 31.4°, corresponding to the (100) and (200) planes of the 3D mixed-halide cubic perovskite phase, respectively. The phase composition of the multi-cation perovskite films processed with different organic molecule-containing anti-solvents was evaluated by XRD measurements. As shown in Figure S4, all films exhibit the characteristic diffraction peaks of the cubic perovskite phase, confirming that the introduction of mCBP during anti-solvent treatment does not alter the fundamental crystal structure. Furthermore, the sharpening of the peaks indicates that mCBP aids in achieving improved crystallinity and phase purity, which is conducive to enhanced optoelectronic performance.

To investigate the effects of mCBP, TPBi, and Bphen on the optical properties and decay behavior of perovskite films, PL and time-resolved photoluminescence (TRPL) measurements were performed, as presented in Figure 1h,i. These measurements provide insights into the fluorescent properties and carrier dynamics of the perovskite films. As shown in Figure 1h, the W/O perovskite film exhibits relatively low PL intensity with an emission peak centered at 484 nm. The introduction of mCBP significantly enhances the PL intensity without shifting the peak position, indicating improved light-emission efficiency. Furthermore, as presented in Figure S5, the absorption spectra of multi-cation perovskite films are nearly unaffected by the treatment with various small organic molecules (mCBP, TPBi, Bphen). The achieved PLQY of 52.31% for the mCBP-treated perovskite film, while representing a substantial 37% enhancement over the W/O one, indicates that non-radiative channels within the film are not fully eliminated. In our multi-cation mixed-halide system (Cs/Rb/K/PEA)Pb(Br/Cl)_3_, compositional heterogeneity is inherent, which can lead to localized low-energy sites or minor secondary phases that act as non-radiative recombination centers or trap states. Furthermore, despite effective passivation of lead-related defects and grain boundaries by mCBP (as supported by XPS and TRPL), other defect types such as halide vacancies and residual disorder at grain boundaries may persist. These contribute to trap-assisted recombination and limit the maximum attainable PLQY. Additionally, while mCBP significantly reduces exciton–phonon coupling (as shown in temperature-dependent PL), some phonon-mediated non-radiative decay pathways remain active at room temperature, especially in polycrystalline films. Future optimization could involve more tailored passivation strategies targeting halide vacancies and further reducing structural disorder to push the PLQY closer to unity. To gain further insight into exciton dynamics and carrier recombination behavior, TRPL measurements were conducted on perovskite films treated with various small organic molecules (mCBP, TPBi, Bphen), as shown in Figure 1i. The fluorescence decay curves were fitted with a bi-exponential function, yielding two characteristic lifetimes with corresponding amplitudes *A*_1_ and *A*_2_: a fast component (*τ*_1_), typically associated with trap-assisted non-radiative recombination, and a slow component (*τ*_2_), attributed to radiative bimolecular recombination. The average lifetime (*τ*_avg_) was calculated to evaluate the overall recombination dynamics, and the fitting quality was assessed using the chi-squared (*χ*^2^) value, as summarized in Table S1. Notably, the mCBP-treated film exhibits the longest average carrier lifetime (39.41 ns), significantly surpassing those of TPBi- (33.68 ns), untreated (29.17 ns), and Bphen-treated (22.72 ns) films. This prolonged carrier lifetime underscores the effectiveness of mCBP in passivating defect states and suppressing non-radiative recombination, thereby promoting more efficient radiative recombination.

**Figure 2:**
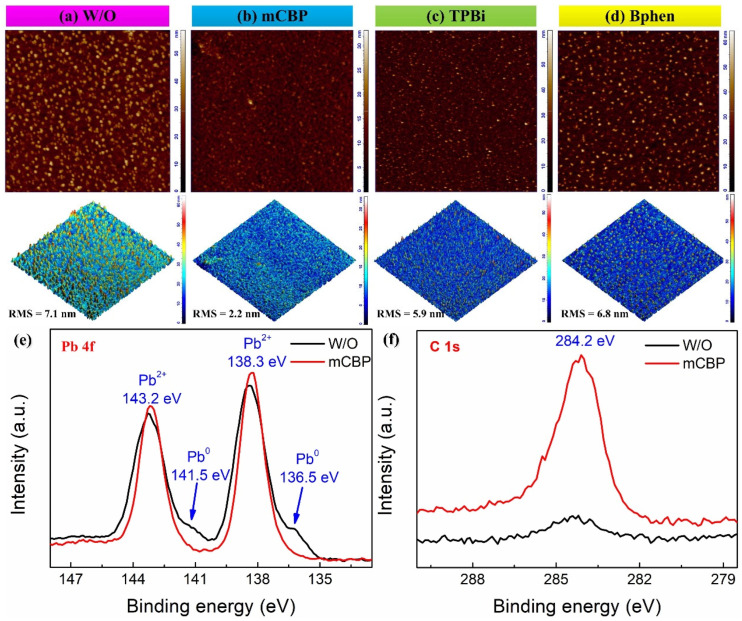
AFM topography and 3D surface morphology (10 × 10 μm^2^ scan area) of perovskite films: (**a**) W/O, and treated with (**b**) mCBP, (**c**) TPBi, and (**d**) Bphen. The root-mean-square (RMS) roughness values are indicated for each film. XPS spectra of W/O and mCBP-treated perovskite films: (**e**) Pb 4f, (**f**) C 1s.

Atomic force microscopy (AFM) was employed to evaluate the surface morphology and roughness of the perovskite films, as illustrated in Figure 2a–d. The W/O perovskite film (Figure 2a) displays a rough and non-uniform surface with prominent irregularities and protrusions, yielding a root mean square (RMS) roughness of 7.1 nm. Such roughness can lead to pinhole formation and hinder charge transport, thereby limiting device efficiency. In stark contrast, the mCBP-treated perovskite film (Figure 2b) exhibits a markedly smoother and more homogeneous surface with well-defined crystal domains, and the RMS roughness is significantly reduced to 2.2 nm. This improvement is crucial for minimizing non-radiative recombination centers and facilitating efficient carrier transport. The TPBi-treated perovskite film (Figure 2c) shows a moderately improved surface compared to the W/O sample, with more uniform grain distribution, though some irregularities remain. The RMS roughness of 5.9 nm indicates partial smoothing, albeit less effective than with mCBP. Similarly, the Bphen-treated perovskite film (Figure 2d) exhibits a somewhat refined morphology with fewer defects relative to the untreated film, yet minor rough areas persist, and the RMS roughness of 6.8 nm reflects only modest improvement. In summary, AFM analysis confirms that mCBP substantially enhances the smoothness and uniformity of perovskite films, offering the most pronounced morphological improvement among the tested additives. While TPBi and Bphen also contribute to surface refinement, their effects are less significant. These morphological enhancements are vital for achieving high-performance PeLEDs, as smoother films reduce defect states, improve charge injection and transport, and boost both device stability and light-emission efficiency. The results underscore the essential role of organic additives, particularly mCBP, in optimizing the surface morphology of perovskite films for advanced optoelectronic applications. To investigate the effect of mCBP treatment on the surface properties of multi-cation perovskite films, X-ray photoelectron spectroscopy (XPS) was carried out for the W/O and mCBP-treated perovskite films. As shown in Figure 2e, two additional weak peaks at 136.5 and 141.5 eV associated with metallic Pb disappeared, while the two main peaks at 138.3 and 143.2 eV assigned to the lead bromide/chloride component in perovskite remained almost unchanged, indicating that the cation-π interaction can suppress the formation of metallic Pb and reduce the defect density in the perovskite films. In addition, the higher intensity of C 1s peaks at 284.2 eV derived from more carbon elements further confirmed the doping of the carbon constituting mCBP (Figure 2f).

**Figure 3:**
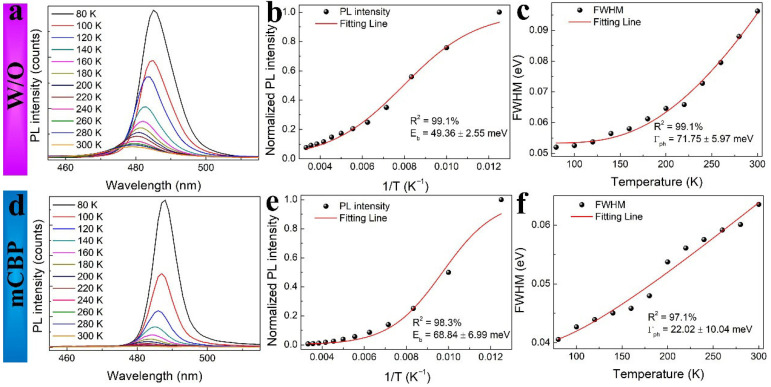
Temperature-dependent photoluminescence (PL) analysis of perovskite films without (**a**–**c**) and with (**d**–**f**) mCBP treatment: (**a**,**d**) PL spectra recorded from 80 K to 300 K. (**b**,**e**) Arrhenius fitting of the integrated PL intensity for extracting the exciton binding energy (*E*_b_). (**c**,**f**) Temperature evolution of the full width at half maximum (FWHM) and the corresponding fitting to extract the phonon coupling coefficient (Γ_ph_).

To investigate the influence of mCBP on the fluorescence properties and thermal stability of perovskite films, we performed temperature-dependent PL measurements and analyzed the full width at half maximum (FWHM) of the emission peaks. As shown in Figure 3a,d, the PL intensity of both untreated (W/O) and mCBP-treated films increases as the temperature decreases, eventually saturating at lower temperatures. The PL intensity was then plotted as a function of inverse temperature (1/*T*) in Figure 3b,e, from which the activation energies (*E*_b_) were extracted. The temperature-dependent PL intensity curves (Figure 3b,e) were fitted using the Arrhenius equation:

IT=I01+Aexp(−EbkBT)

where *I*(*T*) is the PL intensity at temperature *T*, *I*_0_ is the saturated PL intensity at low temperature, *A* is a pre-exponential constant, *E*_b_ is the exciton binding energy, and *k*_B_ is the Boltzmann constant [51]. The extracted *E*_b_ increased from 49.36 ± 2.55 meV for the untreated film to 68.84 ± 6.99 meV for the mCBP-incorporated film. This notable enhancement suggests that mCBP improves exciton stability, likely due to optimized morphological and electronic properties. The temperature dependence of the PL FWHM is illustrated in Figure 3c,f. The data were fitted using Toyozawa’s equation: $\;\Gamma \left(T\right)=A/exp(\Gamma_{ph}/k_{B}T)+\;C$. In the equation, $\Gamma \left(T\right)$ is the FWHM, and *C* is a constant line width at low temperatures due to the absence of phonon broadening. The phonon coupling coefficient (Γ_ph_) was derived from the fitting [52]. The broadening of FWHM with increasing temperature is attributed to enhanced electron-phonon interactions, a common behavior in semiconductors. The exceptionally large reduction in phonon coupling (Γ_ph_: from 71.75 meV to 22.02 meV) reflects multiple synergistic mechanisms. First, mCBP incorporation during crystallization suppresses structural disorder, as evidenced by sharpened XRD peaks (Figure 1g) and reduced surface roughness (AFM, Figure 2b). Reduced disorder directly diminishes phonon scattering centers. Second, mCBP passivates grain boundaries, eliminating localized “soft” phonon modes at defect sites, confirmed by reduced Pb^0^ defects in XPS (Figure 2e). Third, mCBP-induced lattice stiffening reduces the density of low-energy phonon modes available for exciton–phonon coupling. Finally, modified local dielectric screening near organic-inorganic interfaces reduces polarization fluctuations that drive phonon scattering. The correlation between enhanced exciton binding energy and reduced phonon coupling further supports modified dielectric screening as a common origin. This multifunctional role, simultaneously acting as a crystallization modulator, defect passivator, and dielectric modifier, enables mCBP to achieve a large reduction in phonon coupling for blue-emitting perovskite.

Further evidence of defect suppression by mCBP is provided by excitation power-dependent PL measurements using a nanosecond laser. As shown in Figure S6, the face emission spectra of the perovskite films under ns-pulsed laser excitation reveal distinct saturation behaviors. The integrated PL intensity as a function of pump energy, plotted in Figure S7, exhibits a linear dependence at low excitation densities, indicative of exciton-dominated recombination. The saturation pump energy thresholds, extracted from the onset of nonlinear behavior, are 7.87 μJ/cm^2^ for the W/O film, 6.91 μJ/cm^2^ for TPBi-treated, 11.65 μJ/cm^2^ for Bphen-treated, and notably, only 5.82 μJ/cm^2^ for the mCBP-treated film. A lower saturation threshold is often correlated with a reduced density of trap states, as fewer photoexcited carriers are captured by defects before contributing to radiative recombination. The significantly lower saturation energy for the mCBP-incorporated film thus strongly indicates a substantial reduction in defect state density, consistent with its superior TRPL performance and enhanced PLQY. In summary, the combination of prolonged carrier lifetime and lower saturation pump energy threshold unequivocally demonstrates that mCBP effectively minimizes defect-mediated non-radiative losses. These findings highlight the critical role of mCBP in enhancing the optoelectronic quality of multi-cation perovskite films by passivating defects, suppressing exciton–phonon coupling, and improving exciton stability, thereby contributing to the high efficiency and operational stability of the resulting PeLEDs.

**Figure 4:**
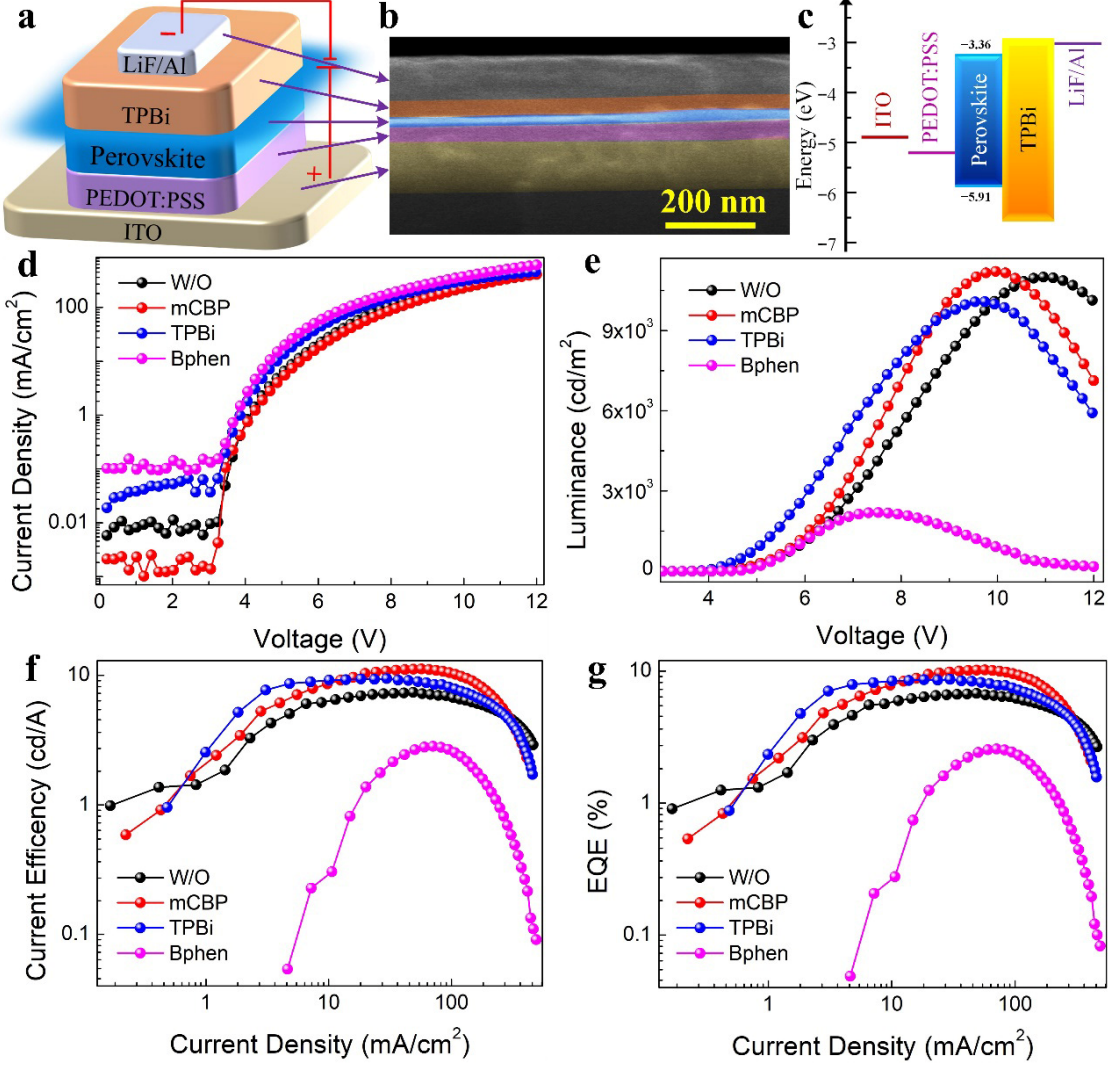
(**a**) Schematic device architecture of the PeLED. (**b**) Cross-sectional SEM image of the complete device stack. (**c**) Energy level diagram of the functional layers. (**d**) Current density–voltage (*J*-*V*) characteristics. (**e**) Luminance–voltage (*L*-*V*) curves. (**f**) Current efficiency versus current density. (**g**) External quantum efficiency (EQE) as a function of current density for PeLEDs based on W/O, mCBP, TPBi, and Bphen-treated perovskite films.

To further investigate the influence of mCBP incorporation on the performance of PeLED devices, we provide a detailed analysis of the device structure illustrated in Figure 4a. The PeLED stack consists of an ITO anode, a PEDOT:PSS hole injection layer, a perovskite emissive layer, a TPBi electron transport layer (ETL), and a LiF/Al cathode. The energy level diagram of the mCBP-treated perovskite film was sketched based on the ultraviolet photoelectron spectroscopy (UPS) (Figure S8) and UV-vis absorption spectroscopy measurements. As shown in Figure 4c, the energy levels of each functional layer are well aligned to promote efficient charge injection and extraction. A cross-sectional SEM image in Figure 4b clearly displays the layered architecture with the emissive layer of approximately 25 nm. Figure 4d compares the current density–voltage (*J*-*V*) characteristics of devices fabricated with different organic additives: W/O, mCBP-, TPBi-, and Bphen-treated perovskite films. Notably, in the low-bias region, the mCBP-based device exhibits the smallest leakage current, while the Bphen-based device shows the highest, indicating superior defect passivation and interfacial quality afforded by mCBP. The mCBP-based device exhibits a steeper increase in current density with voltage, indicating improved charge transport across the perovskite layer. Correspondingly, the luminance–voltage curves in Figure 4e reveal that the mCBP-treated device achieves the highest luminance at lower driving voltages, underscoring its superior electroluminescence efficiency. In contrast, the Bphen-based device shows the lowest luminance output, consistent with its pronounced leakage current, suggesting limited radiative recombination. The current efficiency as a function of current density is plotted in Figure 4f. The mCBP-incorporated device maintains the highest current efficiency across a wide range of current densities, demonstrating excellent operational stability and efficiency retention under high injection conditions. Finally, the EQE versus current density is presented in Figure 4g. The mCBP-modified shy-blue PeLED reaches a peak EQE of 10.2% at an optimal mCBP concentration of 0.3 mg/mL, the highest among all tested configurations. This result confirms that mCBP not only facilitates charge transport and enhances light emission but also effectively suppresses non-radiative recombination losses [4]. Furthermore, the EL spectral stability of the champion mCBP-based device was investigated under different operating voltages, as shown in Figure S9. The EL spectra exhibit a negligible shift in the emission peak at 486 nm, underscoring the excellent spectral stability of our multi-cation perovskite system facilitated by mCBP treatment. The inset of Figure S9 displays digital photographs of the device in operation, emitting uniform and bright sky-blue electroluminescence, which visually corroborates the high quality of the fabricated PeLEDs.

To assess practical viability, we evaluated device operational stability under constant current stress (3.0 mA/cm^2^, N_2_ atmosphere, un-encapsulated). As shown in Figure S10, the mCBP-based device exhibits a T_50_ (time to 50% of initial luminance) lifetime of ~607 s, a significant improvement over the W/O device (389 s). The enhanced stability is attributed to reduced Joule heating from lower leakage current (Figure 4d), suppressed ion migration via grain boundary passivation, and slower defect formation due to more efficient charge transport. Statistical analysis of 20 independently fabricated devices based on mCBP-treated perovskite film confirms the reproducibility of the high luminance, showing an average maximum value of ~10,940 cd m^−2^ (Figure S11), which underscores the reliable and robust brightness enhancement achieved by our mCBP-based strategy. The marked improvement in luminance underscores the critical role of mCBP in optimizing charge injection and transport dynamics. These enhancements are consistent with the observed increase in luminescent efficiency, where mCBP outperforms other interfacial modifiers. By promoting balanced charge recombination and minimizing non-radiative decay pathways, mCBP contributes to both high efficiency and high brightness, key requirements for practical PeLED applications. The origin of this performance enhancement can be traced to the effective suppression of non-radiative triplet exciton quenching at the perovskite/electron-transport layer interface. As established in prior studies, triplet excitons generated within the perovskite emitter can undergo detrimental Dexter energy transfer to adjacent organic layers, such as TPBi with lower triplet energy (T_1_ ≈ 2.6 eV) or Bphen with even lower triplet energy (T_1_ ≈ 2.5 eV), if the triplet energy of the latter is lower than that of the perovskite emitter [4,43,52]. This process serves as a significant energy loss channel in blue PeLEDs. The incorporation of mCBP, which possesses a high triplet energy (T_1_ ≈ 2.9 eV), creates an energetic barrier that confines triplet excitons within the emissive perovskite layer, thereby blocking this parasitic energy transfer pathway. This mechanistic interpretation is strongly supported by the markedly prolonged PL lifetime observed in our mCBP-treated perovskite films and device stacks, consistent with the role of high-triplet-energy interlayers in mitigating exciton quenching [4,43]. Furthermore, the concept of managing triplet excitons at perovskite/organic interfaces has recently been extended to harness their energy in white light-emitting architectures [52], underscoring the broad relevance of triplet exciton management. The perfect correlation between triplet energy hierarchy (T_1_ (mCBP) = 2.9 eV > T_1_ (TPBi) = 2.6 eV > T_1_ (Bphen) = 2.5 eV), and device EQE (10.2% > 8.2% > 5.4%) provides unambiguous validation of triplet management as the governing mechanism. TPBi’s intermediate performance is not a contradiction but rather strong support for our energetically controlled model: its moderate T_1_ provides partial triplet confinement, resulting in intermediate suppression and intermediate EQE. It demonstrates that effective triplet exciton management can be integrated directly into solution processing without requiring vacuum-deposited interlayers. In summary, the incorporation of mCBP into multi-cation perovskite films leads to significant improvements in key device metrics: current density, luminance, current efficiency, and EQE. These gains are attributed to mCBP’s dual function in managing triplet excitons and passivating interfacial defects, as supported by suppressed exciton quenching and improved charge balance. The synergy between mCBP-mediated triplet exciton regulation, multi-cation composition engineering, and defect passivation provides a comprehensive strategy for realizing high-performance blue PeLEDs. This approach opens promising avenues for the development of efficient and stable perovskite-based full-color displays and energy-saving lighting technologies.

## 3. Conclusions

In summary, we demonstrated that incorporating mCBP into multi-cation perovskite films markedly enhances the performance of sky-blue PeLEDs through a scalable, solution-processed approach. The mCBP additive improves film morphology and crystallinity while effectively passivating defects and suppressing triplet exciton quenching, as rigorously validated through transient absorption spectroscopy, magnetic field effect measurements, and Dexter transfer rate analysis. These combined effects lead to enhanced PL properties, optimized charge transport, and overall superior device performance. As a result, the optimized mCBP-based PeLEDs achieve a peak EQE of 10.2%, demonstrating the effectiveness of this solution-based triplet management strategy, and a maximum luminance exceeding 12,000 cd/m^2^. These advancements pave the way for the future application of perovskite-based devices in displays and lighting technologies, offering promising prospects for next-generation energy-efficient and high-performance lighting solutions.

## Data Availability

The original contributions presented in this study are included in the article/Supplementary Materials. Further inquiries can be directed to the corresponding authors.

## Supplementary Materials

The following supporting information can be downloaded at: https://www.mdpi.com/article/10.3390/nano16010004/s1, Figure S1. Photographs of W/O, mCBP-, TPBi-, and Bphen-treated perovskite films under 365 nm UV light irradiation. Figure S2. Elemental mapping and corresponding energy-dispersive X-ray spectroscopy (EDS) analysis of the mCBP-treated multi-cation perovskite film, demonstrating uniform distribution of Cs, Rb, K, Pb, Br, and Cl elements. Figure S3. Photographs of perovskite films with and without mCBP antisolvent treatment under incandescent lamps (top line) and UV lamp illumination at 365 nm (bottom line). Figure S4. XRD patterns of the perovskite films on ITO/glass substrates treated with 0.1, 0.3, and 1.0 mg/mL of mCBP. Figure S5. UV-Vis absorption spectra of the W/O, mCBP-, TPBi-, and Bphen-treated perovskite films. The absorption spectra of perovskite films show similar smooth band edges, revealing that the structure of the perovskite is not affected by the various small organic molecules (mCBP, TPBi, Bphen) treatment. Figure S6. Face emission spectra of the W/O, mCBP-, TPBi-, and Bphen-treated perovskite films pumped by ns pulsed laser. Figure S7. Excitation power-dependent integrated PL intensity of the W/O, mCBP-, TPBi-, and Bphen-treated perovskite films. Figure S8. UPS measurements of the mCBP-treated perovskite film. Figure S9. EL spectra of the champion device based on mCBP-treated multi-cation perovskite film operating under different voltages. The inset shows digital photographs of the champion device in operation. Figure S10. Operational lifetimes of PeLEDs based on the W/O and mCBP-treated perovskite films tested at a constant current density of 3.0 mA cm^−2^. Figure S11. Histogram of maximum luminance for the PeLED device based on mCBP-treated perovskite film. Table S1. Fitted parameters from time-resolved photoluminescence (TRPL) decay curves of perovskite films without and with various small organic molecules (mCBP, TPBi, Bphen) treatment.
