# Supplementary material for: Synergistic Triplet Exciton Management and Interface Engineering for High-Brightness Sky-Blue Multi-Cation Perovskite Light-Emitting Diodes
Source: Nanomaterials (Basel). 2025 Dec 19;16(1):4. doi: 10.3390/nano16010004 (PMC12787356; doi:10.3390/nano16010004)
Supplement: Supplementary file 1 [file nanomaterials-16-00004-s001.zip › nanomaterials-4009941-supplementary.pdf]

Supporting Information

Synergistic Triplet Exciton Management and Interface Engineering for High-Brightness Sky-Blue Multi-Cation Perovskite Light-Emitting Diodes

Fawad Ali [a, #], *Fang Yuan* [a, #, *], *Shuaiqi He* [a], *Peichao Zhu* [a], *Nabeel Israr* [a], *Songting Zhang* [a], *Puyang Wu* [a], *Jiaxin Liang* [a], *Wen Deng* [b], *Zhaoxin Wu* [a, c, *]

[a] Key Laboratory for Physical Electronics and Devices of the Ministry of Education & Shaanxi Key Lab of Information Photonic Technique, School of Electronic Science and Engineering, Xi'an Jiaotong University, Xi'an 710049, China

[b] Journal Editorial Department, Xi'an Jiaotong University, Xi'an 710065 China

[c] Collaborative Innovation Center of Extreme Optics, Shanxi University, Taiyuan 030006, China

* Corresponding Authors:

E-mail addresses: yuanfl21@xjtu.edu.cn (F. Yuan)

zhaoxinwu@mail.xjtu.edu.cn (Z. Wu)

[#]These authors contributed equally to the work.

Experimental section

Materials and chemicals. CsCl (99.99%), RbBr (99.999%), KBr (99.999%), Dimethyl sulfoxide (DMSO) (super dry, 99.8%) and N, N'-Dimethylformamide (DMF) (super dry, 99.8%) were purchased from Alfa Aesar. Lead(II) bromide ($PbBr_2$, 99.99%) were purchased from Aladdin Reagent (Shanghai) Co., Ltd. Phenethylammonium bromide (PEABr, 99%) was obtained from Borun New Material Technology Ltd. 3,3-Di(9H-carbazol-9-yl)biphenyl (mCBP, >99.5%) was acquired from Shanghai Meryer Chemical Technology Co., Ltd. Chloroform ($CHCl_3$, anhydrous, ≥99.8%) were purchased from Sigma-Aldrich. PEDOT:PSS (Clevios PVP AI 4083) was procured from Shanghai Ruofu New Material Technology Co. Ltd. 1,3,5-Tris(1-phenyl-1H-benzimidazol-2-yl)benzene (TPBi, 99%), Bathophenanthroline (Bphen, 99%), and Lithium fluoride (LiF, 99.99%) were purchased from Nichem. All chemicals were used as received without further purification.

Preparation of perovskite solution and film. The precursor solution for the sky-blue multi-cation perovskite was prepared by dissolving $PbBr_2$, CsCl, RbBr, PEABr, and KBr with a molar ratio of 1:0.9:0.5:0.3:0.1 in a mixed solvent of DMSO and DMF (v/v = 7:3). The concentration of Pb^{2+} was maintained at 0.05 mol/L. The solution was stirred vigorously in a nitrogen-filled glovebox at room temperature for at least 4 hours to ensure complete dissolution and homogenization. Prior to spin-coating, both the glass/ITO substrates and the perovskite precursor solution were preheated to 70 °C. The pristine (W/O) perovskite film was fabricated by spin-coating the precursor solution at 1000 rpm for 120 s. During the spin-coating process, 250 μL of chloroform (CF) anti-solvent was swiftly dripped onto the substrate 60 seconds after initiation. The film was subsequently annealed at 80 °C for 20 minutes on a hotplate. For the perovskite films treated with different small organic molecules (mCBP, TPBi, Bphen), the respective compound was dissolved in the chloroform anti-solvent at specified concentrations (e.g., 0.1, 0.3, 1.0 mg/mL) before being applied during the spin-coating step as described above.

Device fabrication. Patterned ITO glass substrates were sequentially cleaned with deionized water, acetone, ethanol, and deionized water in an ultrasonic bath, followed by drying under a nitrogen stream and treatment with UV-ozone for 15 minutes. A filtered aqueous solution of PEDOT:PSS (diluted with deionized water at a volume ratio of 3:1) was spin-coated onto the cleaned ITO substrates at 1500 rpm for 30 s and then annealed at 130 °C for 20 minutes in air. After cooling, the multi-cation perovskite emissive layers were then deposited inside a nitrogen-filled glovebox using the aforementioned procedure. Subsequently, a 40 nm thick TPBi layer, a 1 nm LiF layer, and a 100 nm Al cathode were thermally evaporated onto the perovskite film through a shadow mask under high vacuum ($< 9\times10^{-4}$ Pa). The active area of the devices was defined as 12 mm^2.

Characterization. Steady-state photoluminescence (PL) spectra were measured using a Fluoromax-4 spectrofluorometer (Horiba Jobin Yvon). UV-visible absorption spectra were obtained using a Hitachi U-3010 spectrophotometer. The surface morphology of the perovskite films was examined using a field-emission scanning electron microscope (SEM, Quanta 250, FEI). Atomic force microscopy (AFM) images were acquired in tapping mode using an NT-MDT atomic force microscope. X-ray diffraction (XRD) patterns were recorded on an Empyrean X-ray diffractometer (Malvern Panalytical) with Cu Kα radiation (λ = 1.5406 Å). Time-resolved photoluminescence (TRPL) decay curves were collected using a fluorescence lifetime spectrometer (FLSP920, Edinburgh Instruments) with a pulsed diode laser (λ_{exc} = 375 nm). UV-visible absorption spectra were obtained using a Hitachi U-3010 spectrophotometer. X-ray photoelectron spectroscopy (XPS) and ultraviolet photoelectron spectroscopy (UPS) measurements were performed on an ESCALAB Xi+ spectrometer (Thermo Fisher Scientific) with a monochromatic Al Kα X-ray source and a He I (21.22 eV) UV source, respectively. The photoluminescence quantum yield (PLQY) and temperature-dependent PL measurements were performed using an integrating sphere coupled to the FLSP920 spectrometer from Xi'an Jiaotong University Analysis and Testing Center. The nanosecond pulses were delivered by a frequency-doubled Nd:YAG laser (Surelite I,

Continuum Corp., USA). Face emission spectra were collected by a fiber-optic spectrometer (Ocean Optics SpectraSuite, USB2000). The current density-voltage-luminance (*J*-*V*-*L*) characteristics of the PeLEDs were measured using a Keithley 2602 source meter and a calibrated silicon photodiode. Electroluminescence (EL) spectra were acquired using a PR650 spectroradiometer. All optical and electrical characterizations of the devices were performed at room temperature in a nitrogen atmosphere.

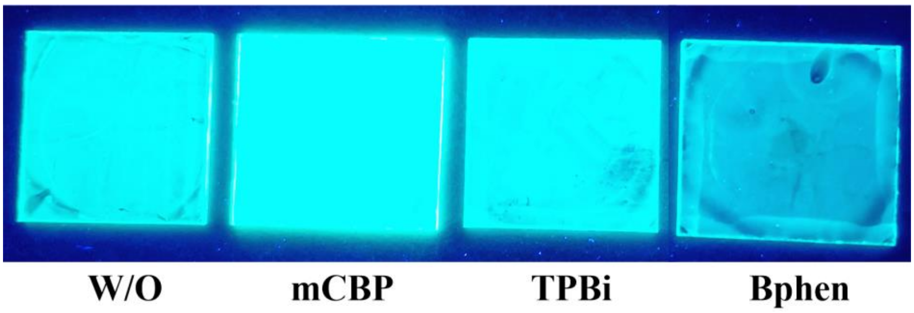

Figure S1. Photographs of W/O, mCBP-, TPBi-, and Bphen-treated perovskite films under 365 nm UV light irradiation.

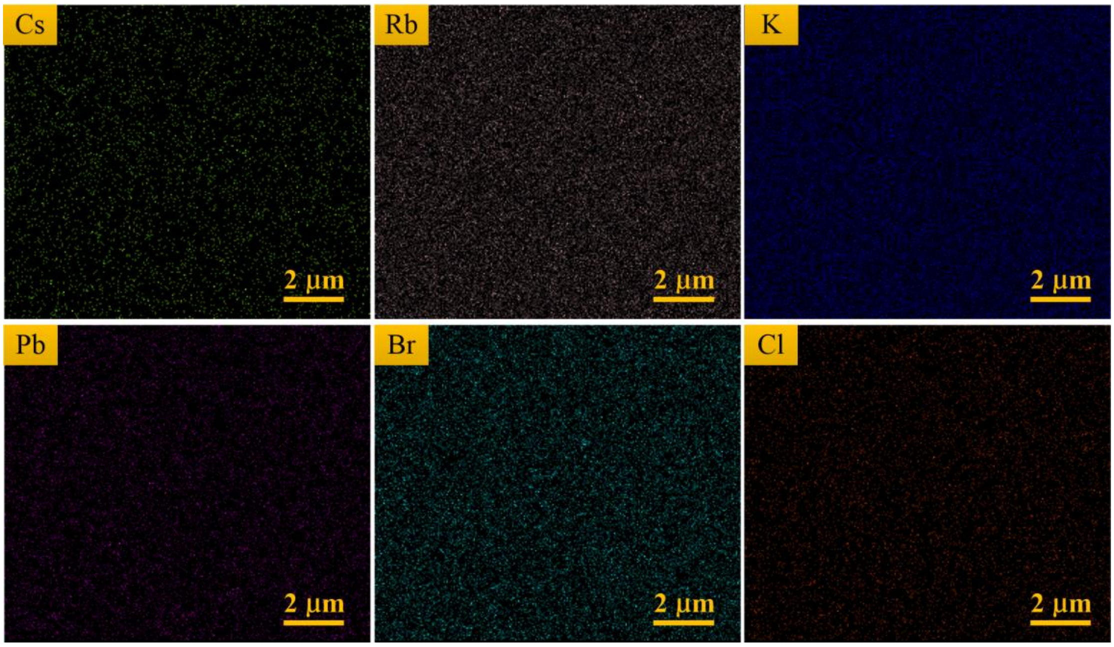

Figure S2. Elemental mapping and corresponding energy-dispersive X-ray spectroscopy (EDS) analysis of the mCBP-treated multi-cation perovskite film, demonstrating uniform distribution of Cs, Rb, K, Pb, Br, and Cl elements.

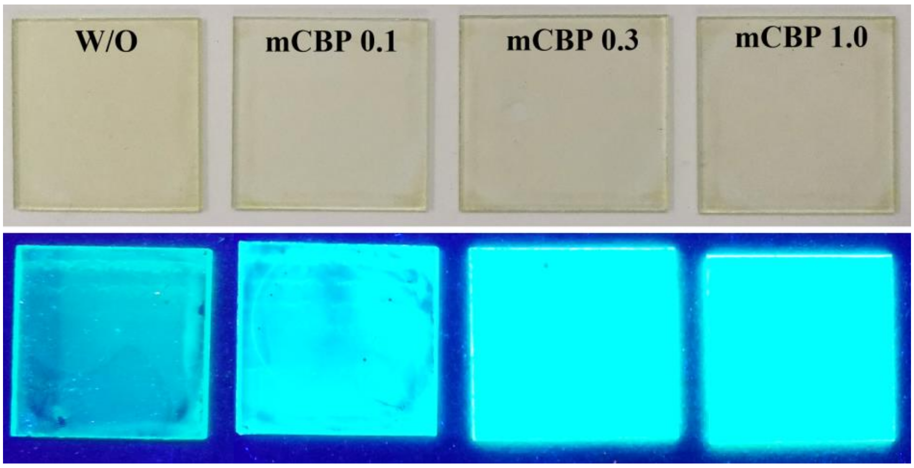

Figure S3. Photographs of perovskite films with and without mCBP antisolvent treatment under incandescent lamps (top line) and UV lamp illumination at 365 nm (bottom line).

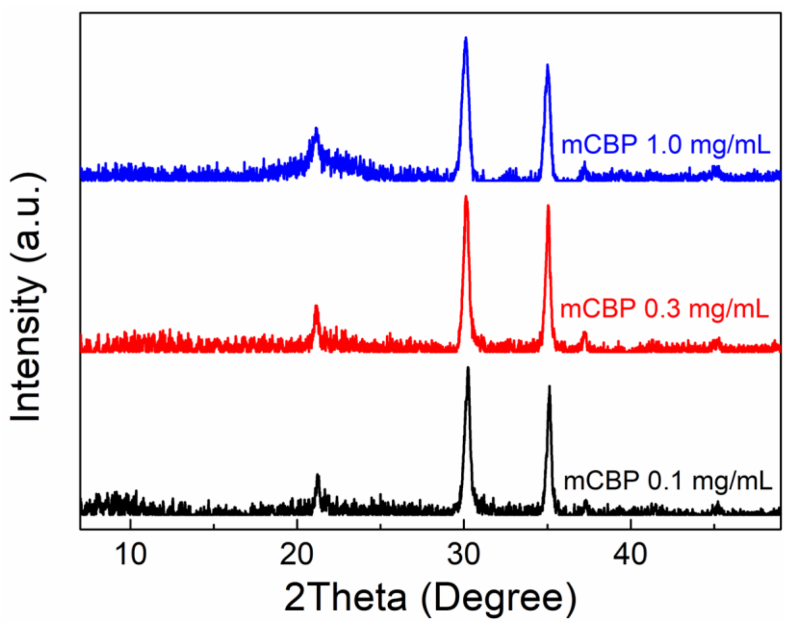

Figure S4. XRD patterns of the perovskite films on ITO/glass substrates treated with 0.1, 0.3, and 1.0 mg/mL of mCBP.

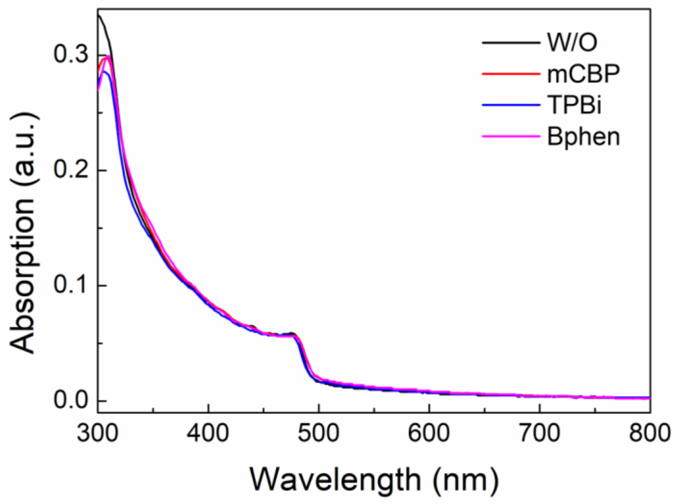

Figure S5. UV-Vis absorption spectra of the W/O, mCBP-, TPBi-, and Bphen-treated perovskite films. The absorption spectra of perovskite films show similar smooth band edges, revealing that the structure of the perovskite is not affected by the various small organic molecules (mCBP, TPBi, Bphen) treatment.

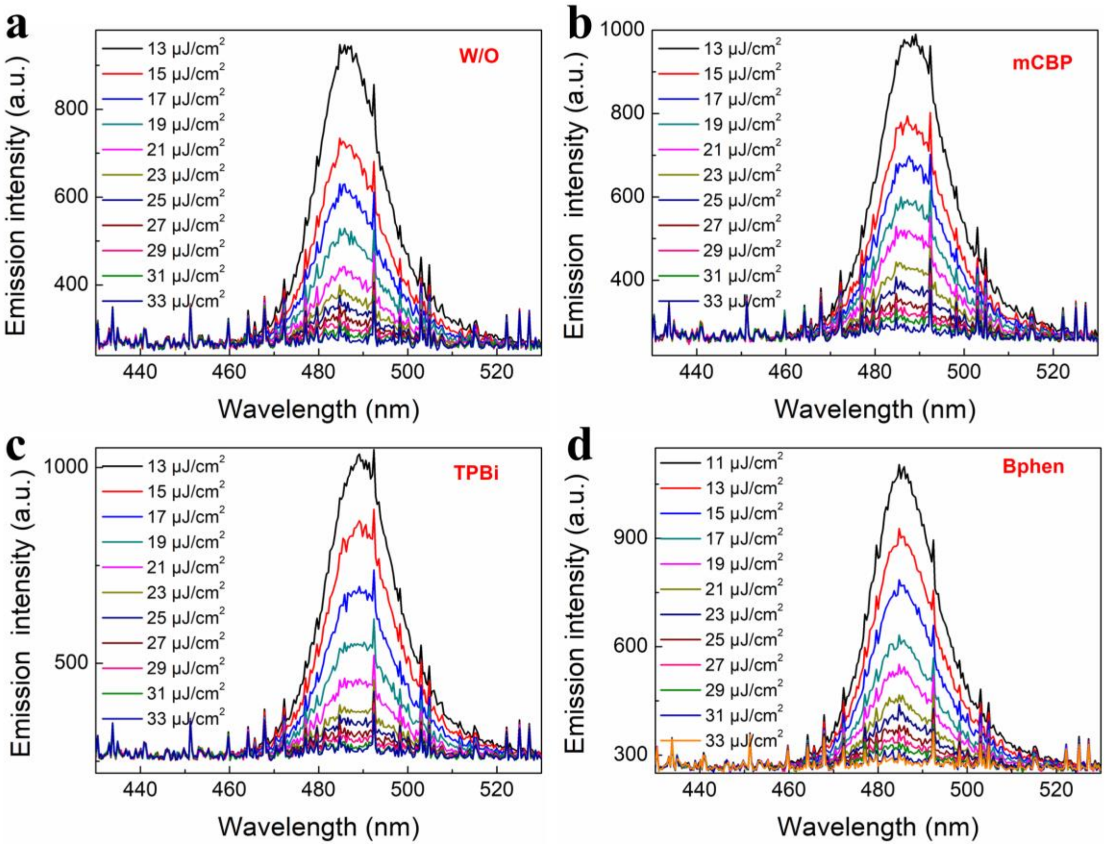

Figure S6. Face emission spectra of the W/O, mCBP-, TPBi-, and Bphen-treated perovskite films pumped by ns pulsed laser.

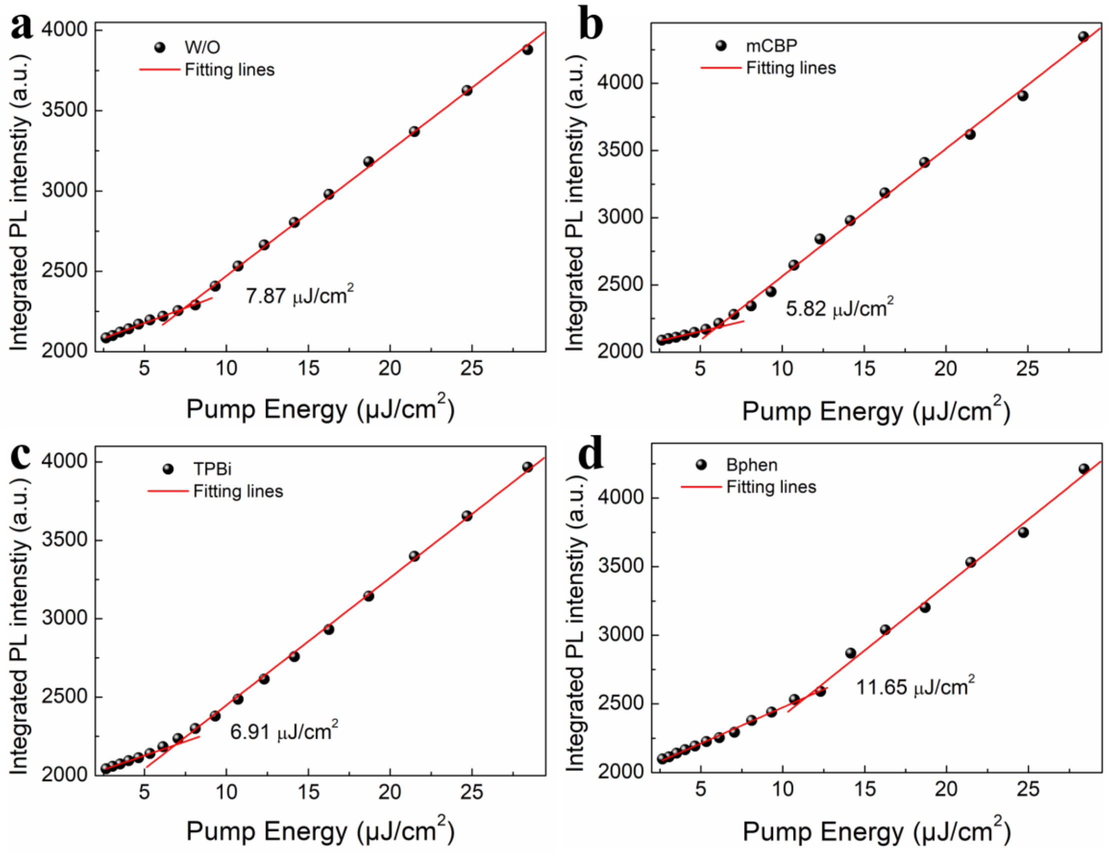

Figure S7. Excitation power-dependent integrated PL intensity of the W/O, mCBP-, TPBi-, and Bphen-treated perovskite films.

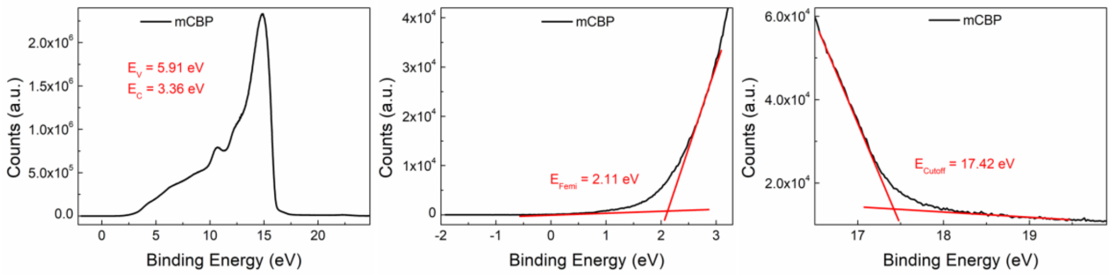

Figure S8. UPS measurements of the mCBP-treated perovskite film.

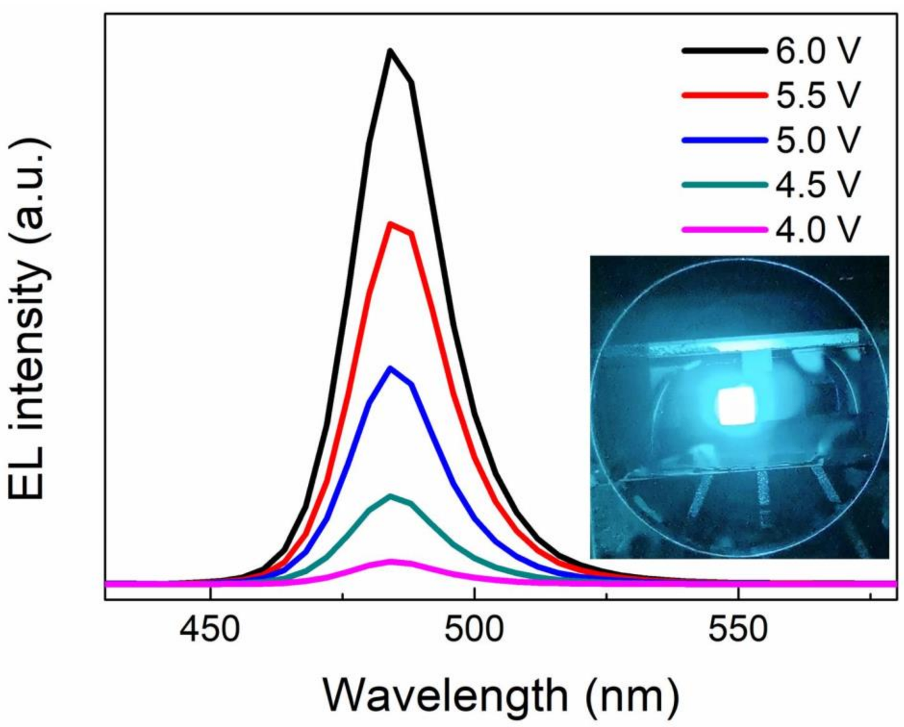

Figure S9. EL spectra of the champion device based on mCBP-treated multi-cation perovskite film operating under different voltages. The inset shows digital photographs of the champion device in operation.

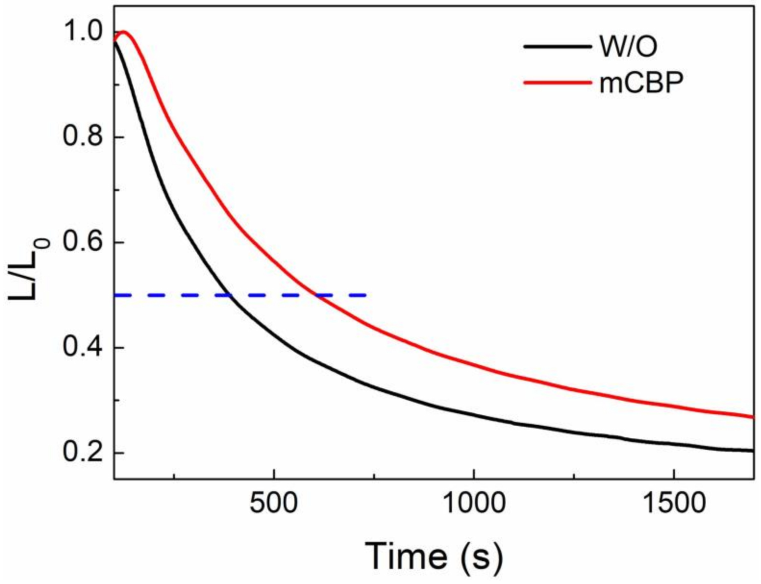

Figure S10. Operational lifetimes of PeLEDs based on the W/O and mCBP-treated perovskite films tested at a constant current density of 3.0 mA cm^{-2}.

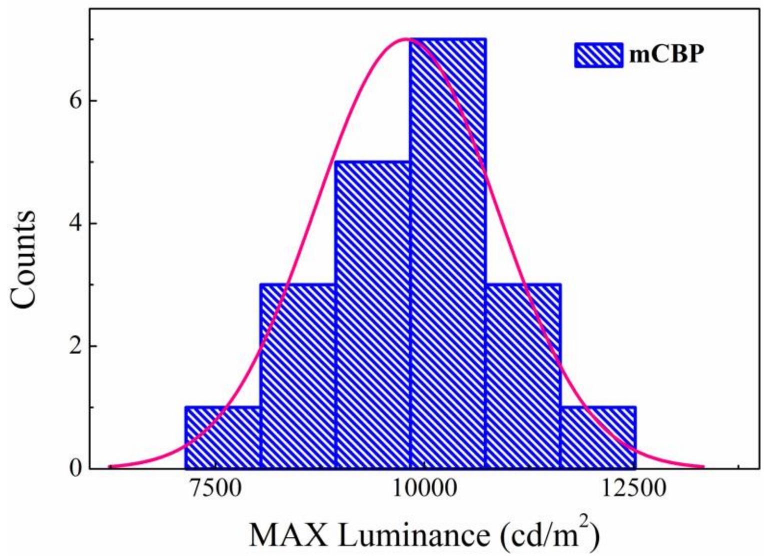

Figure S11. Histogram of maximum luminance for the PeLED device based on mCBP-treated perovskite film.

Table S1. Fitted parameters from time-resolved photoluminescence (TRPL) decay curves of perovskite films without and with various small organic molecules (mCBP, TPBi, Bphen) treatment.

**Sample**	τ_1 **(ns)**	A_1 **(%)**	τ_2 **(ns)**	A_2 **(%)**	χ^2	τ_{avg} **(ns)**
**W/O**	12.97	56.78	50.45	43.22	1.294	29.17
**mCBP**	12.14	54.79	72.46	45.21	1.247	39.41
**TPBi**	13.99	50.61	53.86	49.39	1.181	33.68
**Bphen**	5.45	31.17	3.54	68.83	1.221	22.72

The PL decay curves were fitted to a biexponential function as follows:

$$I(t) = A_1 \exp\left(-\frac{t}{\tau_1}\right) + A_2 \exp\left(-\frac{t}{\tau_2}\right) \quad \text{(S1)}$$

where A_1 and A_2 are the pre-exponential factors, t is PL decay time, τ_1 and τ_2 are fitted lifetimes corresponding to the fast and slow decay components, respectively. The average lifetime τ_{avg}, was calculated using the following equation,

$$\tau_{avg} = \frac{A_1\tau_1 + A_2\tau_2}{A_1 + A_2} \quad \text{(S2)}$$

A tri-exponential decay model was also attempted for the TRPL data analysis. However, the third lifetime component was found to be minor in amplitude and statistically insignificant across samples. Therefore, to maintain consistency with the widely accepted kinetic model for perovskite films and to avoid over-parameterization, the biexponential analysis is presented and discussed herein.
